# Serum PTX3 Levels Correlate with Fibrous Cap Discontinuity and Focal Inflammation in Carotid Vulnerable Plaque: A High-Resolution Magnetic Resonance Vessel Wall Imaging Study

**DOI:** 10.3390/jcm15041452

**Published:** 2026-02-12

**Authors:** Tingting Li, Lu Li, Qingyuan Wang, Yumeng Sun, Jianxiu Lian, Wei Yu

**Affiliations:** 1 Department of Radiology, Beijing Anzhen Hospital, Capital Medical University, 2 Anzhen Road, Chaoyang District, Beijing 100029, China; letdxhc@163.com (T.L.); 13033420084@163.com (L.L.); sym1997928@163.com (Y.S.); 2 Department of Vascular Surgery, Beijing Anzhen Hospital, Capital Medical University, 2 Anzhen Road, Chaoyang District, Beijing 100029, China; 13426336686@163.com; 3 Philips Healthcare, Beijing 100006, China; natalie.lian@philips.com

**Keywords:** PTX3, carotid vulnerable plaque, atherosclerosis, high-resolution magnetic resonance vessel wall imaging, vascular imaging

## Abstract

**Background**: Pentraxin 3 (PTX3) is a well-established inflammatory biomarker with significant implications in the pathogenesis and prognostic assessment of cardiovascular diseases. This study aimed to investigate the potential value of serum PTX3 as a circulating biomarker when combined with the high-resolution magnetic resonance vessel wall imaging (HR-VWI) for identifying carotid vulnerable plaque (CVP) and its specific vulnerable features. **Methods**: This prospective cross-sectional study enrolled 86 patients with carotid atherosclerosis who underwent HR-VWI. Patients were classified into CVP and non-carotid vulnerable plaque (NCVP) groups, and CVP was divided into unilateral carotid vulnerable plaque (UCVP) and bilateral carotid vulnerable plaque (BCVP). CVP was defined as meeting ≥ 1 major criteria: lipid-rich necrotic core (LRNC) maximum area percentage > 40% combined with thin fibrous cap (FC), intraplaque hemorrhage (IPH), FC discontinuity, and focal inflammation. Multivariate logistic regression analysis identified factors influencing CVP and the formation of specific vulnerable features. **Results**: Serum PTX3 levels progressively increased across the NCVP, UCVP, and BCVP groups (median 638.23, 858.52, 1113.62 pg/mL; *p* < 0.001). PTX3 independently predicted the presence of CVP (OR = 2.88; 95% CI: 1.16–8.91; *p* = 0.039) and specific vulnerable features, including FC discontinuity (OR = 2.47; 95% CI: 1.32–4.63; *p* = 0.005) and focal inflammation (OR = 2.25; 95% CI: 1.18–4.32; *p* = 0.014) with high diagnostic performance for these conditions. PTX3 levels exhibited a moderate positive correlation with the number of vulnerable features (r = 0.610, *p* < 0.01). **Conclusions**: Elevated serum PTX3 levels were significantly associated with HR-VWI-defined carotid plaque vulnerability and its severity, serving as a reliable circulating biomarker for identifying FC discontinuity and focal inflammation.

## 1. Introduction

Carotid atherosclerosis is a major cause of ischemic stroke primarily through two pathological mechanisms: plaque ruptures which induce thrombus formation with consequent distal embolism and progressive vascular occlusions precipitating hemodynamic insufficiency [1,2]. Emerging evidence indicates that plaque vulnerability, rather than stenosis severity, serves as the critical determinant of ischemic event risk. Therefore, accurately identifying carotid vulnerable plaque (CVP) is the key to the diagnosis and treatment of ischemic stroke [3,4].

High-resolution magnetic resonance vessel wall imaging (HR-VWI) has emerged as the reference standard for carotid artery wall assessment [5], enabling comprehensive quantification of stenosis severity and plaque burden while simultaneously permitting non-invasive characterization of CVP features, particularly lipid-rich necrotic core (LRNC) and intraplaque hemorrhage (IPH) [6]. Contrast-enhanced T1-weighted imaging (CE-T1WI) and magnetic resonance angiography (CE-MRA) after gadolinium (Gd) administration significantly improves diagnostic sensitivity to vulnerable features such as thin or ruptured fibrous cap (FC), ulceration, and inflammation [7,8].

Circulating biomarkers offer a cost-effective alternative permitting dynamic monitoring of plaque vulnerability. Emerging evidence suggests that elevations in circulating biomarker levels may originate from either de novo pathological plaque formation or destabilization of pre-existing plaques, and thus indicate a high risk of stroke [9]. Multiple circulating biomarkers have been associated with carotid plaque vulnerability. For instance, Lei et al. demonstrated that an elevated ratio of thyroid-stimulating hormones to high-density lipoprotein cholesterol (HDL-C) ratio exhibits predictive value for identifying CVP, particularly those containing LRNC [10]. Evidence has suggested that high expression levels of oxidized low-density lipoprotein cholesterol (oxLDL-C) in both plaques and plasma correlate with plaque instability and increased rupture propensity [11]. Elevated plasma lipoprotein(a) [Lp(a)] levels are associated with the degree of carotid artery stenosis and plaque vulnerability, particularly in terms of IPH, LRNC, and FC status [12]. Moreover, Matrix Metalloproteinase-9 (MMP-9) levels are higher in CVP than in stable plaques [13].

Inflammation is recognized as a critical mediator in atherosclerotic plaque initiation, progression, rupture, and subsequent thrombosis. Pentraxin 3 (PTX3), a prototypical member of the long pentraxin family, is locally synthesized and released by multiple cell types within atherosclerotic lesions, including monocyte-derived macrophages, endothelial cells, vascular smooth muscle cells, fibroblasts, dendritic cells, and adipocytes. Unlike C-reactive protein (CRP) reflecting systemic inflammation, PTX3 provides a more precise characterization of focal inflammatory activity at plaque sites [14,15]. Previous studies have demonstrated associations between elevated circulating and intraplaque PTX3 levels with carotid plaque vulnerability [16]. Furthermore, plasma PTX3 concentrations correlate with carotid stenosis prevalence and severity in ischemic stroke patients [17]. However, the qualitative and quantitative relationships between PTX3 and distinct CVP components remain unelucidated. Therefore, this study utilizes HR-VWI to evaluate carotid plaques with the aim of determining whether PTX3 can serve as a reliable circulating biomarker for predicting carotid plaque vulnerability and specific vulnerable characteristics.

## 2. Materials and Methods

### 2.1. Patient Selection Method

This prospective cross-sectional study consecutively enrolled patients who underwent initial carotid HR-VWI at Beijing Anzhen Hospital, Capital Medical University, from April 2024 to August 2025. All participants were interviewed during hospitalization and provided written informed consent. The study protocol adhered to the principles of the Declaration of Helsinki and was approved by the Institutional Ethics Committee (Approval No. KS2024101).

The inclusion criteria were: (I) age ≥ 18 years; (II) presence of carotid atherosclerotic plaque confirmed by ultrasonography; (III) completion of carotid HR-VWI; and (IV) availability of comprehensive medical records. The exclusion criteria were: (I) severe cardiovascular diseases, hematologic disease, or hepatic and renal insufficiency; (II) active severe infections, chronic or systemic inflammation, or recent use of anti-inflammatory medications within 2 weeks; (III) history of carotid artery stenting or endarterectomy; and (IV) contraindications to magnetic resonance imaging (MRI) or suboptimal HR-VWI image quality.

### 2.2. Baseline Characteristics Collection and PTX3 Measurement

Clinical biomarkers were systematically recorded, which included: age, gender, and body mass index (BMI); cerebrovascular disease (defined as a history of stroke or lacunar infarction confirmed by MRI or medical records), hypertension, dyslipidemia, coronary artery disease (CAD, defined as coronary artery stenosis ≥ 50% confirmed by coronary computed tomography angiography), and diabetes mellitus; medication use of statins, antihypertensive drugs, and antiplatelet agents; and smoking status and alcohol consumption.

For circulating biomarkers analyses, fasting venous blood samples were obtained to measure Lp(a), homocysteine (Hcy), high-sensitivity CRP (hs-CRP), triglycerides (TG), total cholesterol (TC), HDL-C, LDL-C, small dense low-density lipoprotein (sdLDL), non-high-density lipoprotein (nonHDL), remnant-like particle cholesterol (RLP-C), D-dimer, and B-type natriuretic peptide (BNP).

The blood samples were stored in the Clinical Biological Material Resource Center of Beijing Anzhen Hospital, Capital Medical University. Whole blood samples were first collected in clot activator tubes and incubated at 2–6 °C for 30 min to promote clot formation. The tubes were subsequently centrifuged at 2000× *g* for 10 min at 4 °C to separate serum. Aliquoted serum samples were stored at −80 °C until analysis. Serum PTX3 concentrations were measured in triplicate using a commercial human ELISA kit (LAIZEE Biotech, Wuhan, China; Cat# LEH500-2) according to the manufacturer’s instructions. The assay sensitivity for PTX3 was 0.56 pg/mL, with an intra-assay coefficient of variation of 3.7% and an inter-assay coefficient of variation of 4.1%.

### 2.3. Imaging Protocol

MRI sequences were performed on a 3.0 T MRI scanner (Ingenia CX, Philips Healthcare, Best, The Netherlands), carotid HR-VWI was performed using a 32-channel skull coil and an 8-channel cervical coil (TSImaging Healthcare, Beijing, China).

The scanning sequences involved three-dimensional time-of-flight (3D TOF) MRA, two-dimensional T1-weighted imaging (2D T1WI), two-dimensional T2-weighted imaging (2D T2WI), and two-dimensional simultaneous non-contrast angiography and intraplaque hemorrhage (2D SNAP) sequences. A Gd (0.1 mmol/kg body weight) was administered intravenously via the antecubital vein at 3 mL/s, immediately followed by a 20 mL saline flush. CE-MRA sequences were acquired during the arterial phase, with delayed-phase CE-T1WI performed after a 5 min delay (the details of scan parameters are listed in Table 1). The duration of the trial was approximately 30 min. 3D TOF MRA and CE-MRA were used for the evaluation of stenosis degree. 2D T1WI, 2D T2WI, 2D SNAP, and CE-T1WI were used to evaluate the plaque burden and components within 4 cm of the center of the carotid bifurcation.

### 2.4. Imaging Analysis

All carotid HR-VWI datasets were independently analyzed by two board-certified radiologists with more than 2 years of specialized experience in carotid plaque MRI characterization using dedicated plaque analysis software (Vessel Explorer 2, TSimaging Healthcare, Beijing, China) [18]. Both readers were blinded to all clinical information during the evaluation process. HR-VWI cross-sectional slices were manually contoured to delineate lumen and outer wall boundaries. Wall thickness was derived from the distance between lumen-wall interfaces. Automated plaque burden quantification incorporated lumen area (LA), wall area (WA), vessel area (VA), and wall thickness (WT) measurements, with a normalized wall index [NWI = WA/(WA + LA) × 100%]. Software-derived measurements included mean, maximum, and minimum values for all cross-sectional parameters, along with mean lumen, wall, and vessel volumetric indices.

**Figure 1:**
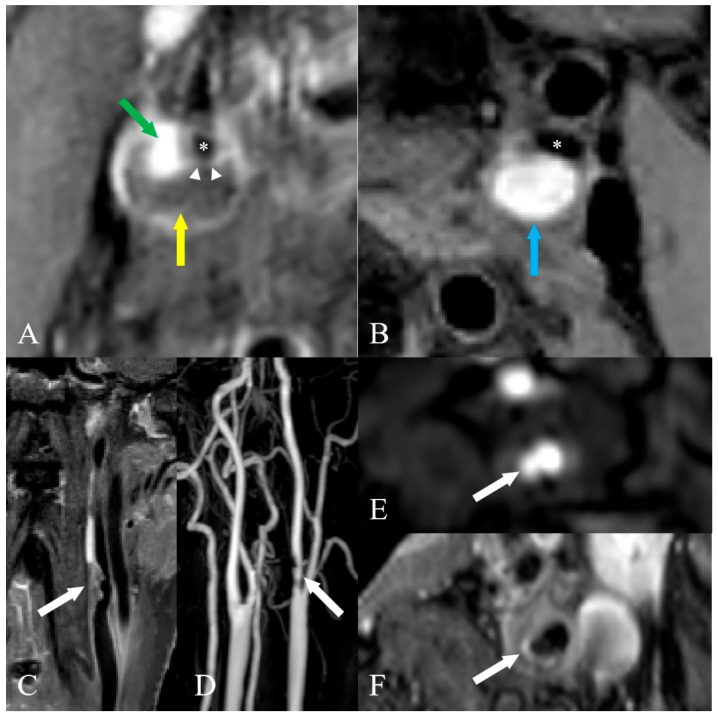
Representative examples of vulnerable features of carotid plaques. (**A**). CE-T1WI demonstrates severe stenosis at the origin of the right internal carotid artery (white *). Focal inflammation (green arrow) within the LRNC (yellow arrow). The FC (white short arrow) shows uneven thickness. (**B**). SNAP reveals severe stenosis at the origin of the left internal carotid artery (white *). A hyperintense area suggests extensive IPH (blue arrow). (**C**–**F**). Severe stenosis at the origin of the left internal carotid artery with FC discontinuity (ruptured FC, ulceration). CE-MRA reveals ulcerated stenosis (white arrow, (**C**)). Coronal VISTA (white arrow, (**D**)), axial 3D TOF (white arrow, (**E**)), and axial CE-T1WI (white arrow, (**F**)) demonstrate plaque surface ulceration. CE-T1WI, contrast-enhanced T1-weighted imaging; SNAP, simultaneous non-contrast angiography and intraplaque hemorrhage; 3D, three-dimensional; TOF, time of flight; VISTA, volume isotropic turbo spin echo acquisition; LRNC, lipid-rich necrotic core; IPH, intraplaque hemorrhage; FC, fibrous cap.

Based on the established criteria, plaque components were assessed, including calcification, LRNC, IPH, and FC status. In addition, hyperintense signals on CE-T1WI were outlined as focal enhancement and were indicative of focal inflammation with neovascularization. Ruptured FC and ulceration were defined as features of the FC discontinuity. The plaque components on the HR-VWI cross-sectional slices were manually drawn segment by segment. Then, plaque component volumes and maximum area percentages were automatically calculated. Plaques were classified as vulnerable if meeting ≥ 1 major criteria: LRNC maximum area percentage > 40% combined with thin FC, the presence of IPH, FC discontinuity, and focal inflammation (Figure 1) [4,5,19,20,21,22].

### 2.5. Patient Groupings

A total of 91 patients were enrolled in the study initially. According to the exclusion criteria, 5 patients were excluded. 86 patients were finally included in this study for further analysis. All patients underwent bilateral carotid artery evaluation and were divided into two groups with either non-carotid vulnerable plaque (NCVP) or CVP. The CVP group was further divided into unilateral carotid vulnerable plaque (UCVP) and bilateral carotid vulnerable plaque (BCVP).

Patients were stratified into high PTX3 (≥938.34 pg/mL) and low PTX3 (<938.34 pg/mL) subgroups based on mean serum PTX3 concentrations of this study cohort. Subsequent analyses compared clinical, circulating, and imaging biomarkers between these subgroups.

We quantified the total number of vulnerable features in bilateral carotid arteries for each patient. Subsequently, CVP groups were grouped by the median vulnerable feature count (3 features), allowing evaluation of the association between PTX3 levels and vulnerability severity.

To investigate the contributing factors underlying the formation of vulnerable features, patients were stratified into groups based on the presence or absence of specific vulnerable features.

### 2.6. Statistical Analysis

We used the Shapiro–Wilk test to assess the normality of continuous variables. Quantitative data that conformed to a normal distribution were expressed as mean ± standard deviation, while those that did not conform to a normal distribution were expressed as median (interquartile range). Qualitative data were expressed as percentages.

Two independent sample *t*-tests and Mann–Whitney U-tests were used to compare normal and abnormal distributions of continuous variables. Chi-square tests were used for comparisons of categorical variables. The Kruskal–Wallis test and Dunn–Bonferroni post hoc analysis were used to contrast the variations between the three independent groups (BCVP group, UCVP group, and NCVP group). Spearman’s rank correlation analysis was used to compare the relationship between PTX3 levels and the number of vulnerable features. Multivariate logistic regression analysis (*p* < 0.05) was used to identify independent predictors of CVP presence (CVP vs. NCVP) and specific vulnerable features occurrence, while also computing adjusted odds ratios (OR) and 95% confidence intervals (CI). The area under the receiver operating characteristic (ROC) curve (AUC) was calculated, and diagnostic efficacy was evaluated by sensitivity and specificity. A two-tailed *p* < 0.05 was considered statistically significant. All statistical analyses were performed using SPSS 27.0 software.

## 3. Results

### 3.1. Patient Baseline Characteristics Based on CVP

Among the 86 enrolled patients, 67 (77.9%) exhibited CVP, comprising 37 unilateral (UCVP) and 30 bilateral (BCVP) cases, while the remaining 19 (22.1%) showed no CVP.

Table 2 shows the clinical and circulating biomarkers tests of the patients in the CVP subgroups and NCVP group. Compared to the NCVP controls, the CVP group had a significantly higher proportion of males (92.5% vs. 73.7%; *p* = 0.038), cerebrovascular disease (55.2% vs. 15.8%; *p* = 0.002), and CAD (62.7% vs. 31.6%; *p* = 0.016), along with elevated median levels of Lp(a) (37.90 vs. 14.60 nmol/L; *p* = 0.012) and PTX3 (942.09 vs. 638.23 pg/mL; *p* < 0.001).

Subgroup analysis revealed progressive increases from the NCVP to UCVP to BCVP groups in the prevalence of cerebrovascular disease (15.8%, 48.6%, 63.3%; *p* = 0.005) and CAD (31.6%, 51.4%, 76.7%; *p* = 0.006), as well as in the median levels of Lp(a) (14.60, 35.70, 48.25 mg/dL; *p* = 0.025) and PTX3 (638.23, 858.52, 1113.62 pg/mL; *p* < 0.001). The D-dimer level in the BCVP group was significantly higher than UCVP group (median 142.00 vs. 81.00, *p* = 0.023).

### 3.2. Patient Baseline and Imaging Characteristics Based on PTX3

Table 3 presents the clinical and circulating biomarkers tests stratified by PTX3 levels. The high PTX3 group (*n* = 38) had a significantly higher prevalence of CAD (73.7% vs. 41.7%, *p* = 0.003) compared to the low PTX3 group (*n* = 48). Additionally, the high PTX3 group showed significantly lower levels of TC (3.37 ± 0.919 vs. 3.89 ± 0.736 mmol/L, *p* = 0.006), HDL-C (median 1.00 vs. 1.06 mmol/L, *p* = 0.019), LDL-C (median 1.69 vs. 1.96 mmol/L, *p* = 0.007), and sdLDL (median 0.51 vs. 0.59 mmol/L, *p* = 0.036), as well as higher BNP levels (median 62.50 vs. 30.00, pg/mL *p* = 0.005).

The high PTX3 group demonstrated significantly greater NWI (63.82 ± 4.725% vs. 60.75 ± 6.066%, *p* = 0.010) and higher prevalence of CVP (92.7% vs. 66.7%, *p* = 0.005) and FC discontinuity (68.4% vs. 20.8%, *p* < 0.001). Quantitative analyses revealed a larger maximum percentage area of focal inflammation (8.38 ± 9.391% vs. 4.83 ± 8.507%, *p* = 0.002) and higher focal inflammation volume (21.34 ± 29.880 vs. 12.39 ± 22.049 mm^3^, *p* = 0.007) in the high PTX3 group (Table 4).

### 3.3. Association of PTX3 Levels with Vulnerable Features

**Figure 2:**
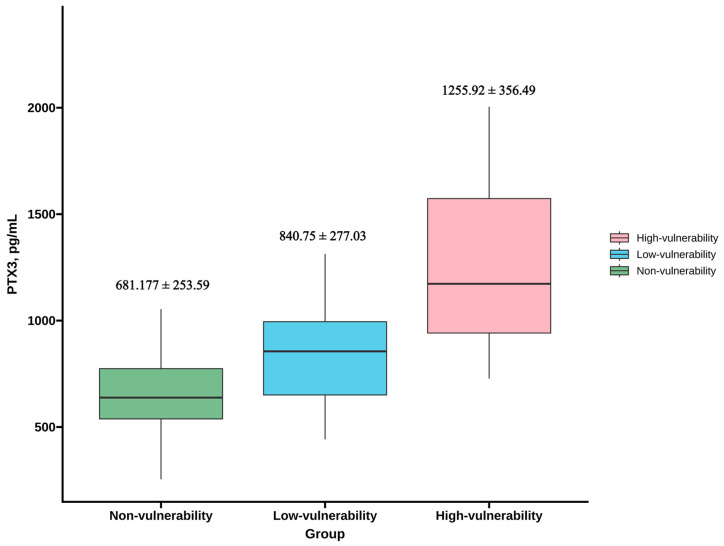
Comparison of serum PTX3 levels among the three groups.

Our analysis evaluated the aforementioned four vulnerable features: LRNC maximum area percentage > 40% combined with thin FC, the presence of IPH, FC discontinuity, and focal inflammation. Analysis of 86 patients revealed that bilateral carotid arteries exhibited up to seven vulnerable features. Using a median threshold of three vulnerable features, patients were stratified into three groups: Group 1 (non-vulnerability, *n* = 19), Group 2 (low-vulnerability: ≤3 features, *n* = 39), and Group 3 (high-vulnerability: >3 features, *n* = 28). As shown in Figure 2, PTX3 differences showed a trend between Group 1 vs. 2 (*p* = 0.062), as well as were significant differences between Group 1 vs. 3 (*p* < 0.01), and Group 2 vs. 3 (*p* < 0.01). Spearman’s correlation analysis demonstrated a moderate positive association between PTX3 levels and the number of vulnerable features (*r* = 0.610, *p* < 0.01).

### 3.4. Analysis of Influencing Factors of CVP Presence

Multivariate regression analysis revealed that cerebrovascular disease (adjusted OR = 4.50; 95% CI: 1.16–22.54; *p* = 0.041) and PTX3 (adjusted OR = 2.88; 95% CI: 1.16–8.91; *p* = 0.039) significantly increased the risk of the presence of CVP (Table 5).

### 3.5. Diagnostic Efficacy of PTX3 in CVP

**Figure 3:**
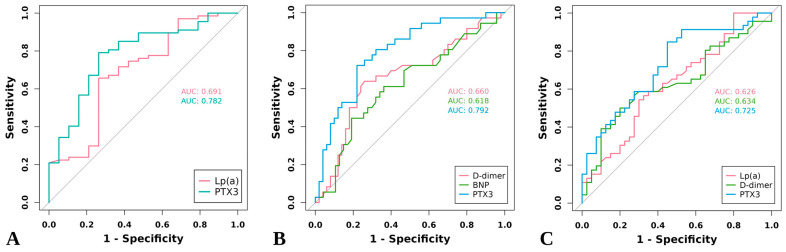
The ROC curves of PTX3 and other circulating biomarkers in predicting (**A**) CVP, (**B**) FC discontinuity, and (**C**) focal inflammation. ROC, receiver operating characteristic; AUC, area under the curve; other abbreviations as in Table 5.

PTX3 demonstrated superior diagnostic efficacy for CVP detection with an AUC of 0.782 (95% CI: 0.662–0.901), significantly outperforming Lp(a) (AUC = 0.691, 95% CI: 0.545–0.836) (Figure 3A). At the optimal cutoff of 723.79 pg/mL, PTX3 achieved 79.1% sensitivity with 73.7% specificity.

### 3.6. Analysis of Influencing Factors of Clinical and Circulating Biomarkers on Specific Vulnerable Features

Preliminary analyses revealed significantly higher FC discontinuity and focal inflammation levels in high PTX3 group. To investigate associated clinical and circulating biomarkers, we stratified CVPs into FC discontinuity-associated CVP (*n* = 36) and focal inflammation-associated CVP (*n* = 46). Biomarkers showing significant differences (*p* < 0.05) or marginal associations in univariable analysis (*p* < 0.1) were included in multivariable analysis when compared to control groups without FC discontinuity or focal inflammation.

In Table 5, the presence of CAD independently predicted FC discontinuity (adjusted OR = 5.88; 95% CI: 1.77–19.56; *p* = 0.004). Elevated PTX3 levels independently increased risks for both FC discontinuity (adjusted OR = 2.47; 95% CI: 1.32–4.63; *p* = 0.005) and focal inflammation (adjusted OR = 2.25; 95% CI: 1.18–4.32; *p* = 0.014).

### 3.7. Diagnostic Efficacy of PTX3 in Specific Vulnerable Features

PTX3 demonstrated strong predictive capacity for FC discontinuity (AUC = 0.792, 95% CI: 0.695–0.888), which significantly outperformed D-dimer (AUC = 0.660, 95% CI: 0.539–0.781) and BNP (AUC = 0.618, 95% CI: 0.494–0.742) (Figure 3B). In the detection of focal inflammation, PTX3 also maintained superior diagnostic performance (AUC = 0.725, 95% CI: 0.618–0.832) when compared to Lp(a) (AUC = 0.626, 95% CI: 0.507–0.745) and D-dimer (AUC = 0.634, 95% CI: 0.515–0.753) (Figure 3C).

## 4. Discussion

In this study, we observed a progressive increase in the prevalence of cerebrovascular disease, CAD, and levels of Lp(a) and PTX3 across the NCVP, UCVP, and BCVP groups. Patients with high PTX3 levels exhibited not only an adverse lipid profile (low HDL-C) and cardiac involvement (high CAD prevalence and elevated BNP), but also heightened carotid plaque vulnerability, including a greater plaque burden (elevated NWI), a higher prevalence of CVP, and more frequent FC discontinuity and focal inflammation. PTX3 positively correlated with the number of bilateral vulnerable features and was an independent predictor of CVP, particularly affecting FC discontinuity and focal inflammation. Moreover, PTX3 outperformed conventional biomarkers in predicting CVP and its specific features.

PTX3 is an inflammatory marker implicated in various cardiovascular pathologies including atherosclerosis, stroke, acute coronary syndrome, acute myocardial infarction, and heart failure [23,24]. Substantial evidence has associated elevated serum PTX3 levels with CAD severity, as higher concentrations can correlate with both increased coronary stenosis severity and the number of affected vessels [25]. Yi et al. [17] identified PTX3 as an independent risk factor for carotid atherosclerosis development and stenosis severity, showing a positive association with the degree of carotid narrowing. Shindo et al. [16] further linked elevated systemic and local PTX3 levels to CVP, which was defined based on an MRI signal intensity ratio > 1.8. To strengthen this link, our study employed HR-VWI to perform a qualitative and quantitative analysis of plaque, thereby providing a more reliable and pathologically grounded classification system. Using this refined criterion, we not only confirmed that PTX3 levels correlated with the number of vulnerable features but also demonstrated its superior predictive value for CVP compared to Lp(a), aligning with prior CAD studies [25]. Furthermore, levels of Lp(a), hs-CRP, and LDL-C showed a rising trend among the NCVP, UCVP, and BCVP subgroups, whereas HDL-C exhibited a declining trend. Although these trends did not consistently reach statistical significance in our analysis, their relationships with carotid plaques vulnerability have all been confirmed in previous studies [12,26,27,28]. Future studies will include more populations to further compare the predictive abilities of different circulating biomarkers for CVP.

However, it is still unclear whether PTX3 can reflect the specific vulnerable features of CVP. This study investigated correlations between serum PTX3 levels and HR-VWI-defined vulnerable features, revealing significant associations between elevated PTX3 concentrations and FC discontinuity and focal inflammation, but no associations with LRNC and IPH were found. Koga et al. [29], using optical coherence tomography to examine coronary plaques, demonstrated that PTX3 levels were significantly elevated in patients with thin-cap fibroatheroma, showing a strong negative correlation with FC thickness. Kimura et al. [30] further identified a dose–response relationship between PTX3 levels and plaque rupture incidence. Pathological analyses by Savchenko et al. indicated that PTX3 was overexpressed in macrophage-infiltrated regions of ruptured plaques, accelerating FC degradation through enhanced MMP-9 expression [31]. In our study, PTX3 exhibited unique predictive value for FC discontinuity that complemented previous research. Given that FC discontinuity is an independent risk factor for ischemic stroke, and that PTX3 levels have been independently associated with stroke severity and post-stroke mortality, PTX3 may hold promise for identifying high-risk plaques prior to FC rupture [32,33,34,35]. This capability could address the limitations of conventional imaging in detecting early plaque instability, offering a novel biomarker for stroke risk stratification and monitoring.

Our study also revealed that elevated PTX3 levels can be associated with focal inflammation. Millon et al. [20] analyzed imaging and pathological findings from 69 carotid plaques and demonstrated that Gd-enhanced regions exhibited 97% neovascularization, 87% macrophage infiltration, and 80% loose matrix (LM) presence. Gd enhancement in plaques primarily reflects focal inflammatory activity and matrix remodeling, with mechanisms including: neovascular endothelial dysfunction promoting Gd extravasation; inflammation-driven extracellular matrix degradation prolonging Gd retention; and direct Gd infiltration into plaques through ruptured FC. In atherosclerotic diseases, PTX3 is produced locally at sites of inflammation by endothelial cells, macrophages, and neutrophils, such that its expression is closely tied to the prediction of focal inflammation.

Elevated PTX3 levels were indicative of a high-burden, high-vulnerability plaque phenotype. This was evidenced by an increased NWI, a higher incidence of CVP and FC discontinuity, as well as larger maximum area percentages and volumes of focal inflammation. A positive correlation was observed between PTX3 levels and the number of plaque vulnerable features, underscoring PTX3’s association with plaque vulnerability severity. This result can be explained by the following mechanisms [16,23,36,37,38]: First, inflammatory amplification where PTX3 synergy with TNF-α/IL-1β/IL-6 activates NF-κB and enhances C1q-mediated complement activation. Second, endothelial function impairment results in dual dysregulation of E-selectin/VCAM-1 (leukocyte recruitment) and P-selectin/MMP-1 (nitric oxide suppression). Third, repair inhibition effect where PTX3 binds to fibroblast growth factor 2, impairing vascular smooth muscle and weakening the repair potential of the plaque FC. Fourth, matrix degradation where PTX3 stimulates macrophages to secrete MMPs, which degrade the collagen and extracellular matrix, leading to thinning of the fibrous cap and loosening of the matrix, thereby compromising plaque stability.

Notably, we observed paradoxically lower TC, LDL-C, and sdLDL concentrations in the high PTX3 group despite previous positive correlations [17]. This finding was corroborated by Kimura et al. [30]. This discrepancy can be resolved through cohort-specific characteristics, as patients with surgically indicated moderate-to-severe carotid stenosis receiving intensive lipid-lowering therapy exhibited PTX3–lipid metabolism decoupling, reinforcing PTX3’s role as an independent cardiovascular biomarker. The dynamic changes in PTX3 are closely related to the response to atherosclerotic treatment. Statin therapy can reduce the circulation of PTX3 levels in both CAD and familial hypercholesterolemia populations (plaque stability improvement) [39,40], while telmisartan-treated CAD patients exhibited selective plasma PTX3 reduction without altering hs-CRP (local anti-inflammatory effect monitoring) [41]. Whether PTX3 can serve as a treatment–response monitoring biomarker for CVP requires further comprehensive investigation.

PTX3 can play a dual role in atherosclerosis. PTX3 modulates polarization of CD163-positive M2 macrophages to suppress localized inflammation [36]. Arterial wall inflammation is exacerbated with significant macrophage infiltration in PTX3 knockout mice [42]. Clinically, analysis of 29 patients with moderate carotid stenosis revealed lower serum PTX3 levels in symptomatic group; however, no correlation emerged in moderate-to-severe stenosis cohorts. This implies that PTX3’s anti-inflammatory and plaque-stabilizing effects may primarily relate to early-stage atherosclerosis [43]. Previous studies have indicated that PTX3 levels correlate with the severity of various cardiovascular diseases, while macrophages can express PTX3 in advanced atherosclerotic lesions, exacerbating vascular injury [23,31,44]. Our study targeted patients with moderate-to-severe stenosis in whom plaques manifested an elevated plaque burden, heightened vulnerability, and significantly upregulated PTX3 levels. These findings position PTX3 as a biomarker that can dynamically reflect inflammatory activity and vascular injury progression. Future research should clarify its stage-specific functions to enable phase-targeted PTX3 interventions across distinct disease stages.

This study had several limitations. First, this study was constrained by a relatively small sample size from a single center with particularly insufficient NCVP groups (*n* = 19), potentially overestimating the diagnostic specificity and threshold of PTX3 for CVP identification. Additionally, pre-admission lipid-lowering therapy in CVP patients may influence PTX3 expression in plaques, necessitating larger-scale studies to precisely quantify PTX3’s predictive efficacy for establishing accurate baseline levels and defining diagnostic thresholds. Second, patients with moderate-to-severe carotid stenosis often exhibited significant cardiac involvement. The current study design cannot fully exclude the confounding effects of coronary plaques on systemic PTX3 levels. Future investigations should incorporate localized PTX3 measurements in carotid plaques preoperatively to clarify correlations between PTX3 production and plaque vulnerability. Third, the prognostic value of PTX3 remains unvalidated, including its ability to predict early postoperative complications (e.g., new ipsilateral ischemic cerebral lesions after carotid stenting) and long-term outcomes (such as in-stent restenosis or stroke recurrence). Multicenter longitudinal cohort studies are warranted to develop PTX3-based dynamic risk stratification models. Fourth, while HR-VWI has demonstrated reliability in characterizing vulnerable plaques, the absence of histopathological validation in this study may limit the definitive interpretation of HR-VWI findings.

## 5. Conclusions

Elevated serum PTX3 levels were significantly associated with the occurrence of CVP and the severity of plaque vulnerability, as assessed by HR-VWI. PTX3 can serve as a reliable circulating predictor of plaque vulnerability, particularly in cases of FC discontinuity and focal inflammation, thereby providing valuable support for clinicians in diagnosis and treatment decision-making.

## Figures and Tables

**Table 1:** Sequence parameters of high-resolution magnetic resonance.

Parameters	3D TOF	2D T1WI/CE-T1WI	2D T2WI	2D SNAP
TE/TR, ms	4/20	10/800	50/4800	6.8/12
FOV, mm^2^	160 × 160	160 × 160	160 × 160	160 × 160
Resolution, mm^2^	0.5 × 0.5	0.5 × 0.5	0.5 × 0.5	0.5 × 0.5
Slice thickness, mm^2^	2	2	2	2
No. of slices	40	20	20	20

3D, three-dimensional; TOF, time of flight; 2D, two-dimensional; T1WI, T1-weighted imaging; CE-T1WI, contrast-enhanced T1-weighted imaging; T2WI, T2-weighted imaging; SNAP, simultaneous non-contrast angiography and intraplaque hemorrhage; TE, echo time; TR, repetition time; FOV, field of view.

**Table 2:** Clinical and circulating biomarkers of patients with CVP subgroups and NCVP group.

Biomarkers	CVP (*n* = 6)		NCVP (*n* = 19)	*p*-Value				
	BCVP (*n* = 30)	UCVP (*n* = 37)		CVP vs. NCVP	K–W Test	Post Hoc		
						UCVP vs. BCVP	BCVP vs. NCVP	UCVP vs. NCVP
Age, years	68.90 ± 6.562	68.03 ± 7.869	65.79 ± 8.973	0.252	0.466	-	-	-
Gender, Male, n (%)	28 (93.3%)	34 (91.9%)	14 (73.7%)	0.038	0.078	-	-	-
BMI, kg/m^2^	24.72 ± 2.653	24.30 ± 3.399	25.45 ± 2.281	0.063	0.140	-	-	-
Cerebrovascular disease, n (%)	19 (63.3%)	18 (48.6%)	3 (15.8%)	0.002	0.005	0.234	0.001	0.020
Hypertension, n (%)	22 (73.3%)	27 (73.0%)	15 (78.9%)	0.769	0.878	-	-	-
Dyslipidemia, n (%)	24 (80.0%)	26 (70.3%)	14 (73.7%)	>0.999	0.663	-	-	-
Coronary artery disease, n (%)	23 (76.7%)	19 (51.4%)	6 (31.6%)	0.016	0.007	0.039	0.002	0.161
Diabetes mellitus, n (%)	15 (50.0%)	11 (29.7%)	8 (42.1%)	0.795	0.237	-	-	-
History of statin use, n (%)	20 (66.7%)	26 (70.3%)	13 (68.4%)	0.984	0.952	-	-	-
History of antihypertensive drug use, n (%)	19 (63.3%)	25 (67.6%)	15 (78.9%)	0.271	0.513	-	-	-
Smoking, n (%)	21 (70.0%)	22 (59.5%)	11 (57.9%)	0.617	0.599	-	-	-
Alcohol consumption, n (%)	15 (50.0%)	14 (37.8%)	4 (21.1%)	0.079	0.130	-	-	-
Lp(a), nmol/L	48.25 (16.60, 86.20)	35.70 (14.50, 68.60)	14.60 (5.80, 63.90)	0.012	0.025	0.329	0.007	0.051
Hcy, umol/L	12.65 (11.20, 14.10)	13.50 (11.20, 15.10)	11.30 (9.00, 15.00)	0.083	0.165	-	-	-
hs-CPR, mg/L	1.25 (0.96, 2.74)	0.85 (0.49, 2.00)	0.80 (0.40, 2.23)	0.338	0.153	-	-	-
TG, mmol/L	1.29 (1.01, 1.68)	1.21 (0.86, 1.74)	1.24 (0.97, 1.87)	0.621	0.793	-	-	-
TC, mmol/L	3.52 ± 0.901	3.75 ± 0.866	3.70 ± 0.784	0.794	0.434	-	-	-
HDL-C, mmol/L	1.00 (0.91, 1.12)	1.06 (0.92, 1.27)	1.03 (0.97, 1.15)	0.673	0.295	-	-	-
LDL-C, mmol/L	1.96 (1.34, 2.19)	1.92 (1.62, 2.50)	1.89 (1.54, 2.44)	0.835	0.610	-	-	-
sdLDL, mmol/L	0.54 (0.43, 0.73)	0.56 (0.42, 0.71)	0.54 (0.36, 0.94)	0.823	0.974	-	-	-
nonHDL, mmol/L	2.44 ± 0.921	2.70 ± 0.870	2.62 ± 0.795	0.887	0.531	-	-	-
RLP-C, mmol/L	0.51 (0.42, 0.74)	0.50 (0.34, 0.69)	0.54 (0.41, 0.68)	0.731	0.762	-	-	-
D-dimer, ng/mL	142.00 (69.00, 206.00)	81.00 (39.00, 149.00)	96.00 (59.00, 116.00)	0.325	0.046	0.023	0.054	0.982
BNP, pg/mL	45.50 (28.00, 84.00)	41.00 (18.00, 97.00)	30.50 (21.00, 68.00)	0.558	0.826	-	-	-
PTX3, pg/mL	1113.62 (862.74, 1573.07)	858.52 (700.66, 1039.77)	638.23 (538.17, 826.21)	<0.001	<0.001	0.003	<0.001	0.022

CVP, carotid vulnerable plaque; NCVP, non-carotid vulnerable plaque; UCVP, unilateral carotid vulnerable plaque; BCVP, bilateral carotid vulnerable plaque; K–W test, Kruskal–Wallis test; BMI, body mass index; Lp(a), lipoprotein(a); Hcy, homocysteine; hs-CRP, high-sensitivity C-reactive protein; TG, triglycerides; TC, total cholesterol; HDL-C, high-density lipoprotein cholesterol; LDL-C, low-density lipoprotein cholesterol; sdLDL, small dense low-density lipoprotein; non-HDL, non-high-density lipoprotein; RLP-C, remnant-like particle cholesterol; BNP, B-type natriuretic peptide; PTX3, pentraxin 3.

**Table 3:** Clinical and circulating biomarkers of patients with low and high PTX3 levels.

Biomarkers	High PTX3 (≥938.34 pg/mL) (*n* = 38)	Low PTX3 (<938.34 pg/mL) (*n* = 48)	*p*-Value
Age, years	69.21 ± 6.683	66.75 ± 8.330	0.132
Gender, Male, n (%)	36 (94.7%)	40 (83.3%)	0.174
BMI, kg/m^2^	24.43 ± 2.989	24.91 ± 2.898	0.448
Cerebrovascular disease, n (%)	22 (57.9%)	18 (37.5%)	0.060
Hypertension, n (%)	29 (76.3%)	35 (72.9%)	0.720
Dyslipidemia, n (%)	30 (78.9%)	34 (70.8%)	0.392
Coronary artery disease, n (%)	28 (73.7%)	20 (41.7%)	0.003
Diabetes mellitus, n (%)	18 (47.4%)	16 (33.3%)	0.186
History of statin use, n (%)	28 (73.7%)	31 (64.6%)	0.366
History of antihypertensive drug use, n (%)	26 (68.4%)	33 (68.8%)	0.974
Smoking, n (%)	22 (57.9%)	32 (66.7%)	0.403
Alcohol consumption, n (%)	16 (42.1%)	17 (35.4%)	0.526
Lp(a), nmol/L	38.50 (11.60, 73.80)	32.70 (12.64, 68.65)	0.617
Hcy, umol/L	13.15 (11.80, 15.30)	12.05 (10.95, 14.80)	0.240
hs-CPR, mg/L	1.15 (0.54, 2.22)	1.12 (0.48, 2.27)	0.821
TG, mmol/L	1.22 (0.92, 1.90)	1.25 (0.95, 1.75)	0.938
TC, mmol/L	3.37 ± 0.919	3.89 ± 0.736	0.006
HDL-C, mmol/L	1.00 (0.84, 1.13)	1.06 (0.98, 1.26)	0.019
LDL-C, mmol/L	1.69 (1.22, 2.19)	1.96 (1.75, 2.52)	0.007
sdLDL, mmol/L	0.51 (0.36, 0.63)	0.59 (0.43, 0.86)	0.036
nonHDL, mmol/L	2.43 ± 0.926	2.72 ± 0.808	0.124
RLP-C, mmol/L	0.50 (0.40, 0.69)	0.52 (0.41, 0.69)	0.976
D-dimer, ng/mL	114.00 (68.00, 206.00)	85.00 (50.50, 146.00)	0.114
BNP, pg/mL	62.50 (32.00, 115.00)	30.00 (19.00, 55.00)	0.005

PTX3, pentraxin 3; other abbreviations as in Table 2.

**Table 4:** Imaging biomarkers of patients with low and high PTX3 levels.

Biomarkers	High PTX3 (≥938.34 pg/mL) (*n* = 38)	Low PTX3 (<938.34 pg/mL) (*n* = 48)	*p*-Value
**Plaque burden, mean value**			
LA, mm^2^	24.41 ± 5.425	27.48 ± 8.175	0.074
WA, mm^2^	42.72 ± 7.949	41.25 ± 10.002	0.277
VA, mm^2^	67.14 ± 11.410	68.73 ± 15.478	0.938
WT, mm	1.65 ± 0.188	1.58 ± 0.231	0.098
NWI, %	63.82 ± 4.725	60.75 ± 6.066	0.010
Lumen volume, mm^3^	987.77 ± 217.827	1096.62 ± 308.413	0.088
Wall volume, mm^3^	1731.44 ± 349.831	1657.58 ± 412.692	0.284
Vessel volume, mm^3^	2719.21 ± 490.934	2754.20 ± 620.443	0.935
CVP, n (%)	35 (92.1%)	32 (66.7%)	0.005
FC discontinuity, n (%)	26 (68.4%)	10 (20.8%)	<0.001
**Maximum area percentage of plaque components**			
Calcification, %	14.44 ± 9.598	15.56 ± 9.804	0.587
LRNC, %	41.03 ± 14.380	39.29 ± 15.074	0.687
IPH, %	14.08 ± 15.026	10.26 ± 14.220	0.157
Focal Inflammation, %	8.38 ± 9.391	4.83 ± 8.507	0.002
**Total volume of plaque components**			
Calcification, mm^3^	177.52 ± 195.350	161.06 ± 170.483	0.928
LRNC, mm^3^	395.52 ± 275.799	392.61 ± 327.957	0.631
IPH, mm^3^	102.08 ± 140.807	79.43 ± 143.984	0.136
Focal Inflammation, mm^3^	21.34 ± 29.880	12.39 ± 22.049	0.007

PTX3, pentraxin 3; LA, lumen area; WA, wall area; VA, vessel area; WT, wall thickness; NWI, normalized wall index; CVP, carotid vulnerable plaque; LRNC, lipid-rich necrotic core; IPH, intraplaque hemorrhage.

**Table 5:** Multivariate logistic regression of CVP group and specific vulnerable features group.

	Indicators	Univariable Analysis			Multivariable Analysis		
		OR	95% CI	*p*-Value	OR	95% CI	*p*-Value
CVP	Gender	4.43	1.13, 17.40	0.033	2.88	0.54, 15.33	0.215
	Cerebrovascular disease	6.58	1.75, 24.72	0.005	4.50	1.06, 19.00	0.041
	Coronary artery disease	3.64	1.23, 10.79	0.020	2.11	0.58, 7.64	0.256
	Lp(a)	3.09	0.97, 9.88	0.057	3.06	0.83, 11.34	0.094
	PTX3	4.39	1.76, 10.95	0.002	2.88	1.05, 7.87	0.039
FC Discontinuity-associated CVP	Coronary artery disease	8.89	3.11, 25.39	<0.001	5.88	1.77, 19.56	0.004
	Alcohol consumption	2.33	0.96, 5.68	0.062	2.00	0.63, 6.35	0.240
	D-dimer	0.95	0.61, 1.50	0.834	0.82	0.45, 1.48	0.511
	BNP	1.46	0.78, 2.73	0.237	1.49	0.85, 2.59	0.164
	PTX3	3.16	1.72, 5.77	<0.001	2.47	1.32, 4.63	0.005
Focal Inflammation-associated CVP	Gender	11.73	1.41, 97.22	0.023	8.34	0.94, 74.11	0.057
	Coronary artery disease	2.80	1.16, 6.74	0.022	1.80	0.65, 4.99	0.260
	Lp(a)	1.62	0.95, 2.74	0.075	1.34	0.75, 2.41	0.322
	D-dimer	5.19	0.84, 32.12	0.076	2.21	0.36, 13.41	0.390
	PTX3	2.69	1.49, 4.83	<0.001	2.25	1.18, 4.32	0.014

CVP, carotid vulnerable plaque; Lp(a), lipoprotein(a); PTX3, pentraxin 3; BNP, B-type natriuretic peptide; OR, odds ratio; CI, confidence interval.

## Data Availability

The data are available from the corresponding author on request.
